# A Critical Review on Vasoactive Nutrients for the Management of Endothelial Dysfunction and Arterial Stiffness in Individuals under Cardiovascular Risk

**DOI:** 10.3390/nu15112618

**Published:** 2023-06-02

**Authors:** Davi Vieira Teixeira da Silva, Diego dos Santos Baião, Cristine Couto Almeida, Vania Margaret Flosi Paschoalin

**Affiliations:** Instituto de Química, Programa de Pós-Graduação em Ciência de Alimentos e Programa de Pós-Graduação em Química, Universidade Federal do Rio de Janeiro, Av. Athos da Silveira Ramos 149, sala 545, Cidade Universitária, Rio de Janeiro 21941-909, RJ, Brazil; davivieiraufrj@gmail.com (D.V.T.d.S.); diegobaiao20@ufrj.br (D.d.S.B.); cristine_almeida@id.uff.br (C.C.A.)

**Keywords:** L-arginine, L-citrulline, Potassium ions, NO bioavailability, dietary interventions, FMD and PWV measures

## Abstract

Pathophysiological conditions such as endothelial dysfunction and arterial stiffness, characterized by low nitric oxide bioavailability, deficient endothelium-dependent vasodilation and heart effort, predispose individuals to atherosclerotic lesions and cardiac events. Nitrate (NO_3_^−^), L-arginine, L-citrulline and potassium (K^+^) can mitigate arterial dysfunction and stiffness by intensifying NO bioavailability. Dietary compounds such as L-arginine, L-citrulline, NO_3_^−^ and K^+^ exert vasoactive effects as demonstrated in clinical interventions by noninvasive flow-mediated vasodilation (FMD) and pulse-wave velocity (PWV) prognostic techniques. Daily L-arginine intakes ranging from 4.5 to 21 g lead to increased FMD and reduced PWV responses. Isolated L-citrulline intake of at least 5.6 g has a better effect compared to watermelon extract, which is only effective on endothelial function when supplemented for longer than 6 weeks and contains at least 6 g of L-citrulline. NO_3_^−^ supplementation employing beetroot at doses greater than 370 mg promotes hemodynamic effects through the NO_3_^−^-NO2-/NO pathway, a well-documented effect. A potassium intake of 1.5 g/day can restore endothelial function and arterial mobility, where decreased vascular tone takes place via ATPase pump/hyperpolarization and natriuresis, leading to muscle relaxation and NO release. These dietary interventions, alone or synergically, can ameliorate endothelial dysfunction and should be considered as adjuvant therapies in cardiovascular diseases.

## 1. Introduction

Blood vessels are constituted of connective tissue, fibroblasts, vascular smooth muscle cells (VSMCs) and endothelial cells (ECs). The endothelium is a semipermeable layer located between the bloodstream and blood vessel wall, comprising a barrier that selectively limits macromolecule movements and guarantees host defense [1]. Endothelial cells, the main endothelium components, play an important role in cardiovascular homeostasis by regulating vascular tone, blood flow, angiogenesis, monocyte/leukocyte adhesion and platelet aggregation [2]. In response to different stimuli, the endothelium maintains the balance between vasoconstriction and vasodilation through the release of both autocrine and paracrine substances, including angiotensin II, endothelin-1, thromboxane A2 and prostacyclin H2, all of which participate in vasoconstriction, while nitric oxide (NO), bradykinin, and hyperpolarizing factors act on vasodilation [3]. The endothelium thus maintains vessel integrity and hemodynamic functions through this self-regulation mechanism [3,4].

Endothelium dysfunctions represent EC failure in maintaining cardiovascular homeostasis, caused by imbalances between endothelium-derived relaxing and contracting factors and leading to deficient vasodilation and low NO synthesis and/or bioavailability in favor of vasoconstrictor elements [5]. Loss of vascular tonus homeostasis can be triggered by several cardiovascular risk factors, such as diabetes mellitus, hypertension, hypercholesterolemia, obesity, aging and chronic smoking, which lead to EC injury and activation [6,7,8,9,10,11,12]. Activated ECs acquire proinflammatory and prothrombotic phenotypes in response to injury, comprising an innate and adaptive immunity mechanism characterized by the overexpression of adhesion molecules and inflammatory cytokines, as well as platelet activation [12]. Endothelial cell activation and dysfunction are important contributors to increased arterial stiffness, atherosclerosis, and cardiovascular events [13,14].

Arterial stiffness is another clinical condition that may contribute to the pathogenesis of atherosclerosis, naturally increasing with age and commonly observed as a complication of endothelial dysfunction in individuals with risk factors for CVD [14,15]. Stiffening of the large arteries can impose extra cardiac muscle effort, leading to heart failure. The pathophysiological mechanisms that lead to arterial stiffness induced by cardiovascular risk factors are numerous, complex and do not result exclusively from endothelial dysfunction, as vessel stiffness can also alter endothelial function [16]. Some of these proposed mechanisms will be briefly discussed below. Aging is associated with lower vessel wall elastin-to-collagen ratios, due to progressive elastic fiber degeneration and increased collagen synthesis [17]. Hyperglycemia induces VSMC proliferation and enhances the formation of advanced glycation products and collagen crosslinking [18]. High blood pressure increases collagen deposition and leads to biomechanical wall fatigue and stiffness in response to repeated pulsatile stress [19]. LDL cholesterol and oxidized-LDL (oxLDL) both increase the production of reactive oxygen species (ROS) via NADPH oxidases (NOX) isoforms such as NOX 1, NOX 2, NOX 4 and NOX 5 expressed in vascular cells, and are the major ROS sources in CVD, resulting in oxidative injury and vessel stiffness [20]. Furthermore, oxLDL triggers VSMC autophagy and apoptosis, with defective autophagy leading to Ca^2+^ homeostasis alterations, enhancing osteogenic differentiation and VSMC calcification [21].

Recent clinical trials, meta-analyses and prospective multicenter observational studies have demonstrated that endothelial dysfunction is closely associated with abnormal arterial stiffness and the development of atherosclerosis, leading to cardiovascular complications. Therefore, early hemodynamic abnormality detection is paramount and depends on the application of reliable and non-invasive techniques, valuable as routine diagnostic procedures. Non-invasive methods, such as flow-mediated vasodilation (FMD) and pulse wave velocity (PWV) measurements, particularly the carotid–femoral (cfPWV) and brachial–ankle (baPWV) pulse wave velocities, which are recognized as indexes of arterial stiffness and the speed at which the arterial pulse propagates along the arterial wall, with cfPWV being considered the gold standard for measuring large artery stiffness, have been employed to assess endothelial function and arterial stiffness, respectively, as cardiovascular event predictors [2,22,23,24].

FMD measures endothelial function through the brachial artery diameter. As NO is the only vasodilatation mediator at this site, FMD brachial artery assessments provide a non-invasive and accurate method to measure endothelial NO production, using imaging to measure arterial dilation following post-occlusive reactive hyperemia. Together, these noninvasive methods can be applied as reliable tools for cardiovascular disease prognostics [25].

Regular fruit and vegetable consumption is associated with decreased CVD. As an example, the Mediterranean diet, characterized by a combination of antioxidant-enriched foods, confers a protective effect against CVD, where dietary compounds may maintain and/or restore cardiovascular homeostasis by increasing NO production, suppressing ROS overproduction and controlling anti-inflammatory activity [26,27]. Therefore, healthy eating patterns can reduce the global burden arising from cardiovascular disease management [28,29].

The current human lifestyle often does not allow for the adoption of healthy diets. Thus, dietary supplementation employing compounds extracted from certain food matrices, as well as new foodstuffs formulated with high concentrations of certain bioactive compounds, may comprise a convenient alternative for human health maintenance.

Several preclinical and clinical trials have highlighted the potential of certain dietary compounds as vasoactive agents, restoring endothelial function through increased NO synthesis, reversing artery stiffness and reinstating endothelial function. Dietary NO_3_^−^, L-arginine, L-citrulline and the mineral potassium (K^+^) are vasoactive compounds acclaimed for their beneficial effects on critical cardiovascular parameters, demonstrated in both animal models and humans [30,31,32,33,34,35,36,37]. L-arginine and NO_3_^−^ are direct precursors of enzymatic and non-enzymatic NO biosynthesis, respectively [38], L-citrulline is a precursor of the endogenous L-arginine synthesis and, at the same time, an NO biosynthesis product, contributing to the de novo L-arginine-NO synthesis [39]; while K^+^ ions are required for normal body fluid volume maintenance, cell membrane potential and the balance between intracellular sodium (Na^+^) and calcium (Ca^2+^) ions, with beneficial effects on vascular smooth muscle relaxation and endothelium-dependent vasodilation [40,41].

In this context, this narrative and critical review describes the mechanisms by which dietary NO_3_^−^, L-arginine, L-citrulline and K^+^, in their pure forms or as a part of rich-food matrices, exert their effects on arterial hemodynamics. Herein, the effects of these nutrients on endothelial dysfunction and arterial stiffness were described at the macrovascular level, i.e., focusing on atherothrombotic large vessels complications that result in myocardial infarction, stroke and peripheral arterial disease, but excluding microvascular complications present primarily as retinopathy, nephropathy and neuropathy from diabetes mellitus. Furthermore, the main findings and benefits evidenced by clinical trials performed on healthy individuals and those at cardiovascular risk following dietary compound supplementation were compiled. The endothelial function and arterial stiffness focusing on FMD and PWV as the main prognostic measures in addition to other indicators such as nitrate and nitrite plasmatic levels (NOx), blood and pulse pressures, and, particularly, the aortic augmentation index (AIx), another arterial stiffness marker, as well as the vascular indices resulting from reactive hyperemia, forming the endothelial function indicator set that reinforces clinical trial findings, were both compiled and discussed.

## 2. Nitrate

Nitrate (NO_3_^−^) is a negatively charged nitric acid salt formed by a single nitrogen atom bound to three oxygen atoms, while nitrite (NO_2_^−^) is a nitrous acid salt formed by a single nitrogen atom bound to two oxygen atoms. Both can be obtained from endogenous and/or exogenous sources [42]. Endogenous NO_3_^−^ and NO_2_^−^ formation occurs by NO metabolism through the L-arginine/NO pathway [3]. Once in the intracellular medium, the amino acid L-arginine undergoes five-electron oxygen-dependent oxidation catalyzed by the nitric oxide synthase enzyme (NOS) and its cofactors, such as calmodulin, Ca^2+^, BH_4_, NAD, NADPH, FAD, FMN and O_2_, forming NO and L-citrulline [43]. In addition, shear stress (the blood flow shear force exerted on endothelial cells) can activate NOS to form NO. Once synthesized, NO is rapidly transformed to NO_2_^−^ by auto-oxidation or through ceruloplasmin, a protein that plays a role in plasma copper transport. The formed NO_2_^−^ can also undergo the action of oxyhemoglobin (oxyHb), generating NO_3_^−^ [44].

**Figure 1:**
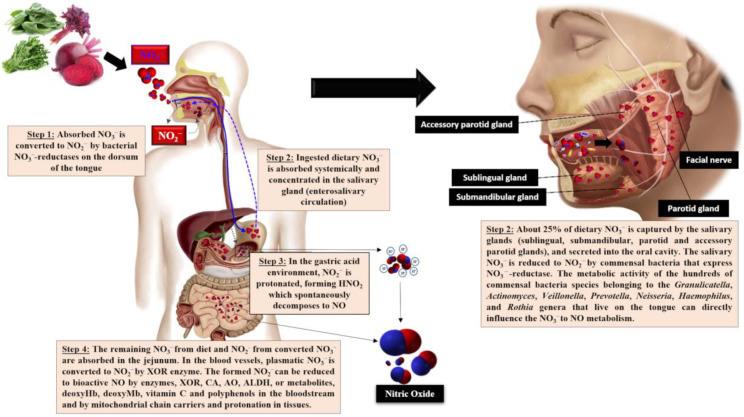
Triggering of the NO_3_^−^-NO_2_^−^/ NO pathway following the ingestion of NO_3_^−^-rich foods. XOR, xanthine oxidoreductase; AO, aldehyde oxidase; ALDH, aldehyde dehydrogenase; deoxyHb, deoxyhemoglobin; deoxyMb, deoxymyoglobin; CA, carbonic anhydrase.

The acquisition of exogenous NO_3_^−^ takes place from drinking water and green and leafy vegetables, in addition to vegetables grown in low-light environments, as NO_3_^−^ is stored and not reduced to form amino acids. Some tubers, mainly beetroot, store high NO_3_^−^ content. In addition, NO_2_^−^ is added to cured meat as a preservative additive [45]. The NO_3_^−^ ingested by the NO_3_^−^-NO_2_^−^/NO pathway is absorbed in the proximal portion of the small intestine, possibly the jejunum, into the bloodstream or tissues, where it accumulates intracellularly as NO_3_^−^. Dietary NO_3_^−^ increases quickly in plasma in about 30 min, peaking at 90 min. About 60% of the absorbed NO_3_^−^ is excreted in urine and 25% is extracted by the salivary glands, concentrated in saliva through the entero-salivary cycle [46]. Concerning the salivary route, NO_3_^−^ in the oral cavity is reduced to NO_2_^−^ by nitrate-reductase expressed by oral commensal bacteria, such as *Streptococcus salivarius*, *S. mitis*, *S. bovis* and *Veillonella* spp., identified as the most prevalent nitrate-reductive microbiota on the tongue that use NO_3_^−^ as a terminal electron acceptor to generate ATP or incorporate it into their biomass [47,48,49]. This NO_2_^−^ mouth generation is sensitive to antibiotics or mouthwash, which can inactivate bacteria, compromising the conversion of NO_3_^−^ to NO_2_^−^ [50]. Furthermore, the metabolic activities of commensal microorganisms that inhabit the oral cavity, such as *Granulicatella* spp., *Actinomyces*, *Prevotella* spp., *Neisseria* spp., *Haemophilus* spp. and those belonging to the *Rothia* genera, can also significantly influence NO_3_^−^ to NO conversion [51,52]. Subsequently, NO_2_^−^ is protonated upon reaching the gastric acid, forming nitrous acid (HNO_2_), which spontaneously decomposes to NO and other bioactive nitrogen oxides, such as nitrogen dioxide (NO_2_), dinitrogen trioxide (N_2_O_3_) and the nitrosonium ion (NO^+^). Furthermore, HNO_2_ may also be decomposed to NO by ascorbic acid and polyphenols [48,49]. In the jejunum, the remaining NO_3_^−^ and NO_2_^−^ are rapidly absorbed into the bloodstream or tissues. Therefore, NO_2_^−^ levels are considerably delayed in circulation, reaching a maximum peak after 2.5–3 h of ingestion [53], the time required for oral cavity NO_3_^−^ to NO_2_^−^ conversion (Figure 1).

Dietary NO_3_^−^ and NO_2_^−^ accumulation occurs by endogenous synthesis through the L-arginine/NO pathway. As mentioned previously, most NO_3_^−^ is lost by renal clearance and a small part is extracted by the salivary glands, concentrating in the saliva, to continue the entero-salivary cycle [54,55,56]. Additionally, a small amount of plasmatic NO_3_^−^ and NO_2_^−^ may be reduced by xanthine oxidoreductase (XOR), which displays similar enzymatic activity to salivary nitrate reductase. Xanthine oxidoreductase catalyzes NO synthesis from the remaining NO_3_^−^ and NO_2_^−^, albeit in the absence of O_2_. Thus, NO can be formed under both hypoxic and ischemic conditions, with increased XOR expression and activity. In addition, NO_2_^−^ can be reduced to NO by deoxyhemoglobin (deoxyHb) and deoxymyoglobin (deoxyMb), especially under low O_2_ levels [49,53]. Other enzymes, such as aldehyde oxidase (AO), aldehyde dehydrogenase (ALDH) and carbonic anhydrase (CA), as well as antioxidant compounds, i.e., vitamin C and polyphenols, display the ability to reduce plasmatic NO_2_^−^ to NO, the bioactive form [54,55].

As NO_2_^−^ is not naturally found in food matrices, due to its instability and quick oxidation to NO_3_^−^, 70 to 80% of NO_2_^−^ exposure originates from food additives mixed with foodstuffs. These compounds are used to improve food taste, color and appearance and prevent food oxidation, as well as the growth of foodborne pathogens and secretion of harmful compounds, such as the botulinum toxin, during meat and baked goods and cereal processing [57]. Thus, plasma NO reflects dietary NO_3_^−^ intake, with 85% originating from vegetables in Western diets, although the content of this anion varies between edible plants from distinct botanical families [50]. Indeed, NO_3_^−^ content in vegetables depends on their genetic background or environmental factors such as atmospheric humidity, temperature, water content and exposure to sunlight and irradiation, as well as agricultural practices, i.e., crop type, fertilization, soil conditions, the use of fertilizers and herbicides, the amounts of available nitrogen and the availability of other nutrients, and, finally, post-harvest conditions, such as transportation and storage conditions [50,58]. The NO_3_^−^ contents in plant organs also differ, classified from the highest to the lowest contents as petiole > leaf > stem > root > tuber > bulb > fruit > seed. Among vegetables considered the richest NO_3_^−^ food sources, beetroot (1300 mg of NO_3_^−^·kg^−1^), arugula (4677 mg of NO_3_^−^·kg^−1^) and spinach (2500 mg of NO_3_^−^·kg^−1^) are the most popular with respect to dietary interventions, all resulting in effective cardiovascular performance improvements estimated through blood pressure decreases and vascular function improvements [49,50,58]. Furthermore, a single serving portion of any of these vegetables contains more NO_3_^−^ than is formed through internal human body processes per day. However, it is important to note that NO_3_^−^ supplementation from leafy greens has been tested only in healthy individuals, and it is unknown whether its effects can be extended to individuals displaying cardiovascular risk factors. Although the protective cardiovascular effects of NO_3_^−^-enriched vegetables have been clearly demonstrated in clinical trials with healthy subjects, the large vegetable serving portions to be ingested to achieve effective NO_3_^−^ plasma concentrations may comprise a limiting factor in ensuring adherence to long-term nutritional interventions [3]. In this regard, the low NO_3_^−^ content in serving portions has been overcome by developing different beetroot formulations that concentrate pharmacological NO_3_^−^ doses in small serving portions of an attractive food product, favoring continuous intake and better adherence to a non-drug strategy therapy in order to improve endothelial function in individuals at cardiovascular risk [3].

However, strict standards regarding the levels of these anions in foods and drinks have been established in the past. Until a decade ago, NO_3_^−^ was considered a toxic compound derived from unfavorable diets, as it was mistakenly associated with the development of certain malignancies, such as metglobinemia (MetHba) and gastric cancer [59,60,61]. Therefore, the Food and Agriculture Organization of the United Nations/World Health Organization (FAO/WHO) defined an acceptable daily intake of 3.7 mg of NO_3_^−^·kg^−1^ of body weight in 1962, the same level adopted by the European Food Safety Authority [62,63]. For a healthy 80 kg adult, this content is the equivalent of ~300 mg NO_3_^−^·day^−1^. However, the adoption of vegetarian diets, in general, increases NO_3_^−^ consumption in 80 kg adults to over 350 mg.day^−1^, well above the stipulated acceptable daily intake [64]. The association between NO_3_^−^ and NO_2_^−^ and MetHba in adults and children, however, has not been proven in the literature [61,65]. Furthermore, several studies have failed to demonstrate a link between dietary NO_3_^−^ and NO_2_^−^ ingestion and the production of N-nitrosamines, carcinogenic compounds that can lead to tumor development [66]. This evidence supports a significant link between cancer and red processed meat, with little knowledge of the effects of vegetables and drinking water available. In this regard, the inorganic NO_3_^−^ and, particularly, inorganic NO_2_^−^ added during meat processing may contribute to cancer development [67]. Nonetheless, the hypothesis that both dietary NO_3_^−^ and NO_2_^−^ from foods, mainly from plant origin, are toxic has been established based merely on conjecture.

Health organizations have established an adequate NO_3_^−^ intake of around 40–185 mg·day^−1^ (1 to 3 mmol·day^−1^) in Europe and 40–100 mg·day^−1^ (1 to 1.6 mmol·day^−1^) in the USA, considering 100% NO_3_^−^ bioavailability following dietary intake [68]. However, considering the role of NO_3_^−^ on cardiovascular system function, none or minimal beneficial hemodynamic and vascular effects have been observed following acute NO_3_^−^ administration or short-period administration for under 14 days [3,58]. Increases in plasmatic NO_3_^−^ levels, from 31 to 150 μM, as well as NO_2_^−^, from 0.23 to 0.40 μM, have been observed, but no improvements were detected in SBP and FMD following 14 days of supplementation with 7.5 mmol NO_3_^−^ from beetroot juice in elderly patients with type 2 diabetes mellitus [69]. No changes in arterial stiffness, assessed by PWV and AIx, or in blood pressure were observed in normotensive individuals after a 7-day intake of 6.4 mmol NO_3_^−^ from green leafy vegetables, although increased plasmatic NO_3_^−^ levels, from 23.4 to 152 μM, and NO_2_^−^, from 2.0 to 8.0 μM, were observed [70]. Furthermore, Bondonno et al. [71] did not observe modifications in domestic BP, and ambulatory 24 h SBP and DBP in hypertensive individuals supplemented for 7 days with 7.0 mmol NO_3_^−^ from beetroot juice, although increased NO synthesis was observed, assessed through NO_3_^−^ and NO_2_^−^ determinations in plasma, urine, and saliva.

On the other hand, when NO_3_^−^ is provided as a chronic dietary supplementation, the beneficial effects on vascular function are more consistents. Kapil et al. [72] observed a decrease in systolic (SBP) and diastolic blood pressure (DBP), decrease in PWV, and an increase and improvement of AIx and FMD, respectively, of hypertensive volunteers after supplementation with 6.4 mmol NO_3_^−^ during 28 days, corresponding to 400 mg/day of beetroot juice. Endothelial function and arterial stiffness improvements and decreased blood pressure were observed simultaneously with increased NO synthesis, estimated by increased NO_3_^−^ plasma levels, from ≈40 to ≈200 μM, as well as higher NO_2_^−^ levels, from ≈0.4 to ≈0.9 µM. Rammos et al. [73] administered 150 µmol·kg^−1^ of NaNO_3_^−^, at least 10.5 mmol for a 70 kg individual, for 28 days to elderly patients presenting moderate CVD risks, also describing increases in average NO_3_^−^ plasma concentrations, varying from 32 to 263 μM, and NO_2_^−^ concentrations, ranging from 0.08 to 0.33 μM, alongside improved FMD and decreased SBP, DBP, PWV and AIx. Recent systematic reviews and meta-analysis studies have evaluated vascular responses to dietary NO_3_^−^ and confirmed BP decreases and endothelial dysfunction amelioration. A systematic review conducted by Bahadoran et al. [74] demonstrated that beetroot juice intake reduces SBP [−3.55 mm Hg; 95% CI: −4.55, −2.54 mm Hg] and DBP [−1.32 mm Hg; 95% CI: −1.97, −0.68 mm Hg]. Furthermore, decreased SBP depends on the amount of beetroot juice intake, where comparisons between 500 mL and 70 mL and 140 mL·day^−1^ indicate −4.78 vs. −2.37 mm Hg decreases. Longer supplementation periods compared to shorter ones (≥14 days vs. <14 days of treatment) led to a −5.11 vs. −2.67 mm Hg decrease. In another systematic review and meta-analysis, randomized controlled trials indicated that NO_3_^−^ supplementation from beetroot juice for longer than 14 days reduced both SBP (−3.55 mm Hg; 95% CI: −4.55, −2.54 mm Hg) and DBP (−1.32 mm Hg; 95% CI: −1.97, −0.68 mm Hg). Furthermore, beneficial dietary NO_3_^−^ effects on endothelial function are associated with dose, age, and body mass index (BMI), where chronic beet juice supplementation improved FMD and endothelium function according to the administered NO_3_^−^ dose (β = 0.04, SE = 0.01, *p* < 0.001), age (β = −0.01, SE = 0.004, *p* = 0.02) and BMI (β = −0.04, SE = 0.02, *p* = 0.05) [75].

Based on the studies already reported and included herein, it can be concluded that to promote the NO formation and the improvement of hemodynamic and vascular parameters, i.e., reversal of both endothelial dysfunction and arterial stiffness in individuals presenting cardiovascular risk factors, the supplementation of NO_3_^−^ should be over 370 mg (6.0 mmol) per day [69,72,76,77]. In addition, endothelial function and hemodynamic parameter improvements, as well as decreased arterial stiffness, following dietary NO_3_^−^ intake, even when administered at high concentrations could be usually achieved if NO_3_^−^ supplementation is extended, comprising chronic ingestion for over 20 days [72,74,75] (Table 1).

## 3. L-Arginine

**Figure 2:**
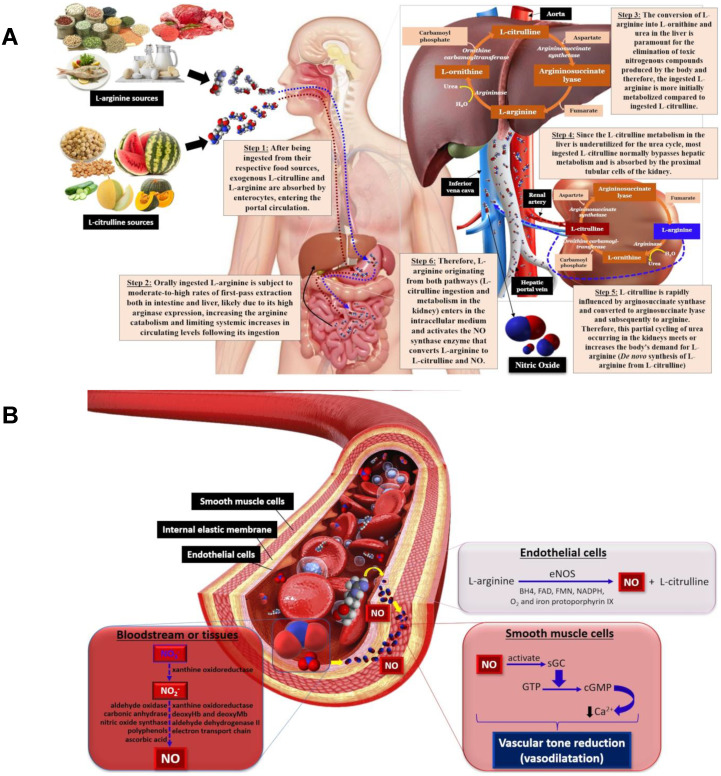
The metabolic pathway for L-arginine and L-citrulline obtained from food sources, urea cycle and NO biosynthesis (**A**); NO biosynthesis from L-arginine by eNOS promotes vasodilation by activating guanylate cyclase to form cGMP that, in turn, decreases Ca^2+^ within smooth muscle cells, diminishing vascular tone and leading to vasodilation (**B**). eNOS—endothelial nitric oxide synthase, BH_4_—tetrahydrobiopterin, FAD—flavin adenine dinucleotide, FMN—flavin mononucleotide, GC—guanylate cyclase, GTP—guanosine triphosphate, cGMP—cyclic guanosine monophosphate, Ca^2+^—calcium ions.

L-arginine (2-amino-5-guanidinopentanoic acid) is a semi-essential cationic amino acid obtained through dietary intake, protein turnover, and/or de novo synthesis from L-citrulline in liver, and from the kidney urea cycle [90]. Oral L-arginine undergoes gastrointestinal and hepatic extractions before reaching portal circulation, where arginases from enterocytes and liver catalyze the hydrolysis of L-arginine into L-ornithine and urea, which limits systemic L-arginine levels (Figure 2A). L-arginine is also biosynthesized in the kidneys through L-citrulline metabolism following the conversion of exogenous L-citrulline (not metabolized in the liver first-pass) to the precursor arginosuccinate, catalyzed by arginosuccinate synthase, and then converted to L-arginine by arginosuccinate lyase in the urea cycle [90].

L-arginine is involved in NO synthesis, and is employed as a substrate for nitric oxide synthase (NOS) class enzymes, comprising neuronal (nNOS), inducible (iNOS) and endothelial (eNOS) isoforms. As mentioned previously, L-arginine, the substrate, adenine dinucleotide phosphate (NADPH) as the electron cofactor, and O_2_ are involved in NO synthesis, forming citrulline and NADP^+^. Tetrahydrobiopterin (BH_4_), flavin adenine dinucleotide (FAD), flavin mononucleotide (FMN) and iron protoporphyrin IX are all cofactors involved in this reaction [91]. Once synthesized, NO diffuses from endothelial cells to smooth muscle cells in blood vessels and activates the soluble guanylate cyclase (GC) enzyme that, in turn, catalyzes the conversion of guanosine triphosphate (GTP) to cyclic guanosine monophosphate (cGMP) and pyrophosphate (PPi) [92]. Subsequently, cGMP decreases intracellular Ca^2+^ concentrations by activating the calcium pump within smooth muscle cells, inducing vasodilation through reduced vascular tone [93,94,95] (Figure 2B).

L-arginine is found in nuts, such as peanuts and walnuts, but also in foods of animal origin, such as meats, poultry, fish and dairy products, providing an intake of about 4.4 g/day of this amino acid in Western diets [96]. Considering that L-arginine is the NO substrate for NO synthesis and its involvement in endothelium-dependent dilatation, the beneficial effects of L-arginine supplementation on endothelial dysfunction and arterial stiffness have attracted attention for some time, aiming to overcome hemodynamic abnormalities and risk factors, thus avoiding cardiovascular events.

In a prospective, double-blind, randomized crossover trial with elderly healthy individuals (age 73.8 ± 2.7 years), the L-arginine supplementation of 16 g/day for 2 weeks increased L-arginine plasma levels from 57.4 ± 5.0 mM to 114.9 ± 11.6 mM, improving endothelial-dependent vasodilation [34]. Similar to aging, smoking represents a harmful condition leading to endothelial dysfunction and decreased NO biosynthesis alongside inflammatory responses caused by endothelium injury, which can be counteracted by L-arginine supplementation. Six grams/day of L-arginine administered for 3 days to 10 healthy smokers in a randomized, placebo-controlled, double-blind, and cross-over clinical trial led to an improvement on FMD baseline and prevented smoking-induced FMD decreases at day 1. The supplementation was, however, unable to sustain this up to day 3. On the other hand, L-arginine decreased both PWV and AIx at days 1 and 3 compared to the placebo [97]. In a similar study, L-arginine supplemented at 21 g/day for 3 days to 12 healthy smokers improved FMD baselines, but did not cease smoking effects, while smoking-induced PWV and AIx increases were inhibited by L-arginine supplementation [98]. L-arginine may, therefore, be considered a very promissory positive effector, able to prevent the arterial stiffness increment associated with smoking behavior. L-arginine also seems to improve endothelial function in subjects presenting coronary artery disease (CAD) [99]. Following an intake of 21 g of L-arginine for 10 days resulted in an FMD improvement by 4.7 ± 1.1 vs. 1.8 ± 0.7% (*p* < 0.04). Similarly, the intake of 10 g of L-arginine by stable CAD patients for a longer period of time of 4 weeks reduced endothelial dysfunction, as demonstrated by changes in FMD diameter, increasing the diameter by 4.87% (*p* < 0.0001) compared to baseline values. The conclusions from that study, however, were limited, due to its open-label clinical trial character, as the placebo group received vitamin C, which is also an active compound [100]. On the other hand, in a similar clinical double-blind and placebo-controlled trial, chronic L-arginine therapy was shown to increase the reactive hyperemia of the forearm blood flow by employing venous occlusion plethysmography in individuals displaying stable CAD for 6 months. The hyperemic flow of the forearm is considered an endothelial-mediated vasodilation marker and has been shown to depend on NO synthesis. The positive correlation between the 6.4 g/day L-arginine supplementation, c-GMP and reactive hyperemia increments reinforces the role of L-arginine in NO synthesis enhancement and consequent endothelial function improvement [101]. Coronary endothelial dysfunction has been proposed to predict the progression of atherosclerotic disease and cardiovascular event rates [102], and, although the clinical trials presented in this review have not evaluated the effect of L-arginine on the coronary artery, the findings presented herein indicate that L-arginine supplementation may comprise a promising alternative against endothelial function impairment in CAD patients and be able to inhibit unfavorable cardiovascular outcomes.

L-arginine intake for 28 days or more has also been shown to protect against others chronic pathologies deleterious to vascular function. The daily ingestion of 8 g L-arginine improved endothelial function in 28 women with polycystic ovary syndrome (PCOS) who were making use of oral contraceptives. PCOS is a pathological condition associated with low NO bioavailability due to its inadequate release, production, or degradation. This results in endothelium-dependent vasodilatation impairment and cardiovascular risks that may be exacerbated by oral contraceptives [103,104]. For the treatment of PCOS, ultrasonographic and Doppler flow evaluations revealed that L-arginine supplementation promoted a significant improvement in brachial artery diameter and pulsatility index at 15 s after reactive hyperemia, compared to the baseline. This effect was not observed in contraceptive plus placebo group. L-arginine supplementation did not lead to any blood pressure variations, whereas the placebo group displayed increases for 24 h, both during the day and during the night. Increased plasma NO_3_^−^ and NO_2_^−^ following L-arginine intake confirmed NO availability and endothelial function improvements. However, the most important finding in this intervention comprised the fact that endothelial function and NO production effects were extended for 6 months following L-arginine supplementation, demonstrating a sustained effect following 28 days of L-arginine administration [104].

Oral administration of 8 g L-arginine to 15 congestive heart failure patients for 60 days led to endothelial function improvements, evaluated through the maximum amplitude time (MAT), total wave time (TT) and MAT/TT ratio obtained through photoplethysmography following forearm blood flow occlusion and reactive hyperemia in comparison to pre-ischemia levels [105]. Photoplethysmography can be used to indirectly evaluate endothelial function in peripheral vessels by sensing vasodilatation in the index finger, since changes in blood flow and pulse wave amplitude are the result of flow-mediated vasodilatation following NO synthesis [106]. Although the dietary intervention was limited due to the low number of patients, the absence of a control group, and the method reflecting only the microvascular status, L-arginine supplementation decreased MAT/TT values to under 30, similar to values observed in healthy individuals, demonstrating its modulation on NO-mediated vasodilation [107]. Likewise, a 12-week supplementation with 9 g of L-arginine was able to circumvent the PWV ≥ 900 cm/s commonly found in chronic kidney disease (CKD) patients. Clinical CKD manifestations increase the risks for CAD, heart failure and cardiac death due to the pathological vascular remodeling, calcification and arterial stiffness that underly kidney disease [107]. The chronic intake of L-arginine promoted a significant increase in NO levels and decreased aortic stiffness, overcoming NO baseline levels and PWV, confirming vascular damage caused by kidney impairment and the effectiveness of L-arginine supplementation in reversing hemodynamic abnormalities in CKD patients, probably caused by a defective L-arginine/NO biosynthesis [108].

Postprandial endothelial dysfunction is a clinically important condition and has been reported following high-fat meals in both healthy subjects and in those with risk factors for CVD. Postprandial endothelial dysfunction has been proposed to be triggered through oxidative stress induced by hypertriglyceridemia, where NO bioavailability is reduced by the superoxide anion (O_2_^−^), resulting in the generation of the highly reactive and cytotoxic peroxynitrite [109,110,111]. In overweight adults with high triglyceride plasma levels and waist circumference measurements, L-arginine supplementation was able to overcome cardiovascular risk factors following 4.5 g supplementation for 4 weeks, where an inhibition of the decrease in postprandial endothelial function induced by a high-fat meal was observed, demonstrated by a 29% reduction in FMD when compared to a 50% reduction in FMD in placebo-treated subjects. In this clinical trial, a 5% increase in reactive hyperemia was also observed, while there was a 49% reduction in placebo treatment [112]. Similarly, a single dose of 15 g of L-arginine attenuated the FMD reduction promoted by high-fat meals compared with a placebo treatment in forty healthy men (from 10.3 ± 1.3 to 9.3 ± 0.9% in the L-arginine supplementation group and from 10.5 ± 1.2% to 6.8 ± 1.4%, in the placebo group) [113]. These findings indicate that L-arginine may increase NO bioavailability and reduce endothelial dysfunction induced by postprandial hypertriglyceridemia.

Notwithstanding the clinical studies performed in recent years (Table 2), recent literature data point to the need for additional clinical trials, establishing adequate L-arginine doses, intervention times, target populations, baselines, and hemodynamic parameters, such as FMD, carotid and brachial–ankle PWV, and plasmatic asymmetric dimethylarginine (ADMA) and NOx (nitrate plus nitrite) levels, which may contribute to knowledge of the effectiveness of L-arginine therapy [114]. On the other hand, other authors suggest that L-arginine may be one of the most important therapeutic molecules for the treatment of cardiovascular disorders [115]. Following the studies reported herein, several clinical trials with doses ranging from 4.5 to 21 g for a minimum of 2 weeks and a maximum of 12 weeks pointed to beneficial outcomes following both acute and chronic L-arginine supplementation on endothelial dysfunction, and therefore, such doses of L-arginine can be considered as a starting recommendation for non-drug therapy in individuals at cardiovascular risk. Although some clinical trials have been cited in the present review [97,98,108], the body of evidence associated with L-arginine artery stiffness effects is still weak, albeit promising against adverse smoking effects.

## 4. L-Citrulline

L-citrulline, a non-essential amino acid not commonly found in proteins, is involved in nitrogen homeostasis and shows promising vascular benefits in promoting endothelial vasodilation. L-citrulline is efficiently converted to L-arginine, the endogenous NO biosynthesis precursor that acts in reducing arterial stiffness, as discussed previously (Figure 2B) [35,116,117]. Dietary L-citrulline passes through the liver to the kidneys, where it is converted to L-arginine via the argininosuccinate biosynthesis in the urea cycle, producing extra NO (Figure 2A). Studies have reported that L-citrulline can indirectly reduce blood pressure by increasing NO biosynthesis, improving arterial function, blood flow and circulation and, thus, reducing the risk of heart disease [118,119].

L-citrulline is found in legumes, fruits, and grains, such as onions, garlic, chickpeas, peanuts, and soy, and the highest concentrations of this amino acid are found in watermelon pulp and rinds in concentrations ranging between 0.7 and 3.6 g.kg^−1^ of fresh weight depending on the type of cultivar [117,120,121]. L-citrulline has been widely commercialized as a supplement, and is available at higher doses than those found in natural foods. Marketed L-citrulline is mainly consumed by athletes to assist in sports performance and muscle mass gain [122,123].

Although L-citrulline was for many years considered simply a metabolic intermediate of little biological interest, studies have confirmed that it increases circulating L-arginine levels more efficiently than L-arginine supplementation, the final product formed during L-citrulline metabolization. A large amount of the ingested L-arginine is degraded during the extensive pre-systemic metabolism by intestinal bacteria and by arginase found in the liver and gut mucosa. In contrast, L-citrulline is preserved during the pre-systemic metabolism, effectively transported across the intestinal luminal membrane and then converted to L-arginine in the kidneys. Subsequently, L-arginine is converted to L-citrulline and NO by eNOS in endothelial cells [35,54,90,119,124]. The absorption characteristics of oral L-citrulline indicate that the use of this amino acid comprises an attractive non-pharmacological approach that may counteract cardiovascular pathophysiological conditions. Clinical trials have been conducted to assess whether oral L-citrulline intake improves endothelial function compared with other strategies to assess the effectiveness of L-citrulline in increasing NO bioavailability following L-arginine supplementation (Table 3).

The endothelial function effects of watermelon ingestion for seven consecutive days were investigated on the basis of FMD measurements. Six healthy overweight/obese adults received 100 kcal serving portions prepared from watermelon pulp, rind, and seeds, while the control group received flour. FMD reactivity assessed in the brachial artery after 7 h intake showed no differences, with the authors ascribing the inconclusive results to a low sample number [125]. When evaluating the supplementation of eleven young adults with 30 g of microencapsulated watermelon rind (MWR) containing 4 g of L-citrulline, improved endothelial function assessed by FMD was observed alongside with increased L-citrulline and L-arginine plasma levels [126].

One hypothesis postulate that postprandial hyperglycemia and acute hyperlipidemia both induce endothelial dysfunction as measured by FMD throughout oxidative stress induction, since free radicals quench NO, disrupting endothelial-dependent vasodilation [110]. In a randomized, placebo-controlled, double-blind, crossover trial, 17 healthy young adults from 21 to 25 years old, 6 males and 11 females, were supplemented with 500 mL of watermelon juice for 2 weeks and underwent an oral glucose tolerance test followed by postprandial FMD, to evaluate endothelial function following hyperglycemia induction and L-citrulline supplementation effects. Although no significant effects were observed on plasma L-citrulline and L-arginine, the latter showed a tendency to increase compared to placebo and the postprandial FMD area AUC was higher after juice supplementation when compared with the placebo group (838 ± 459% vs. 90 min compared with 539 ± 278% vs. 90 min). [127]. In this way, supplementation with L-citrulline, as a precursor of L-arginine and consequently of nitric oxide, seems to have the potential to attenuate the endothelial dysfunction induced by high glucose levels, but more studies need to be carried out.

Another randomized, double-blind, placebo-controlled trial study was performed to evaluate the effects of watermelon juice on vascular health, albeit in 21 healthy postmenopausal women. Subjects were randomized to consume two 360 mL servings of 100% watermelon juice ingested daily or an isocaloric placebo for 4 weeks. Vascular function assessments included pulse pressure, PWV, 24 h ambulatory BP, and FMD. In contrast to the findings of previous clinical trials in younger adults, the watermelon juice supplementation did not affect vascular parameters compared to the placebo, indicating that a 720 mL dosage/day of watermelon juice is insufficient to alter serum L-arginine in postmenopausal women, possibly explaining the unchanged vascular function [128]. On the other hand, watermelon supplementation (L-citrulline/L-arginine 6 g/d) to twelve obese, hypertensive postmenopausal women for 6 weeks significantly lowered baPWV, aortic SBP and DBP compared to placebo [129], which suggests that the vascular effects of watermelon L-citrulline may be more pronounced when the individual has some cardiovascular risk factor.

Following randomized crossover studies, watermelon intake enriched in L-citrulline promoted aortic blood pressure and arterial function amelioration in hypertensive individuals. The pilot study assessed nine participants, four men and five women aged 54 ± 3 years, who were diagnosed with pre-hypertension and consumed 2.7 g/day of watermelon or placebo for 6 weeks. Both the AIx and AIx adjusted for an HR of 75 beats/min (AIx 75) decreased in the watermelon-supplemented group (−6.0 ± 3% and −4.0 ± 2%, respectively). At the same time, no PWV carotid–femoral or reflected wave (T*r*) transit time effects were noted [130]. In another study, carotid AIx (cAIx) measures were performed instead of aortic AIx, since the former can more precisely reflect the central AIx. After 6 weeks of watermelon supplementation containing 6 g of L-citrulline/L-arginine administered daily, decreased cAlx values (−8.8 ± 2.6%) were observed in 14 obese middle-aged adults presenting prehypertension or stage 1 hypertension, reflecting artery endothelium function improvements [119].

Acute ingestion of L-citrulline (3 g) effectively increases the availability of L-arginine and NO in both young and elderly adults with heart failure. L-citrulline supplementation increased NO synthesis 10-fold, but was ineffective at promoting endothelium-mediated vasodilation in the two CVD subject groups. These results imply that other factors besides NO may play a role in vascular dysfunction. In addition, longer-term L-arginine or L-citrulline supplementation is required to reverse peripheral vascular function impairment in older adults with heart failure. Younger adults are more sensitive to the acute ingestion of L-citrulline compared to the elderly, due to the efficacy in converting this compound to L-arginine. Older adults present a higher L-arginine to ornithine conversion rate via arginase in the urea cycle, illustrating NO synthesis differenced with aging [131].

In another study, twenty-five sedentary hypertensive postmenopausal women aged 50 to 74 were randomized for 4 weeks and administered L-citrulline (10 g) or a placebo. Plasma L-arginine, FMD, cfPWV, brachial and aortic BP were evaluated, and the findings suggest that 4 weeks of L-citrulline supplementation were effective at improving serum L-arginine levels, FMD, aortic DBP and MAP compared to the placebo. In contrast, cfPWV and brachial BP were not altered. Serum L-arginine levels increased after 4 weeks of L-citrulline supplementation (12.7 ± 2.4 μM/L) compared to the placebo (−1.8 ± 1.7 μM/L), with a concomitant FMD increase (1.4 ± 2.0%) compared to the baseline and placebo (−0.5 ± 1.7%). Thus, L-citrulline supplementation may comprise a viable therapeutic strategy against apparent vascular complications in hypertensive postmenopausal women [132].

The short-term effects of L-citrulline extract supplementation on arterial stiffness were investigated in 15 healthy subjects aged 58.3 ± 4.4. The volunteers that received 5.6 g/day of L-citrulline (n = 8) or a placebo (n = 7) for 7 days exhibited baPWV decreases, but no differences in blood pressure were detected between the two groups, and no correlation was observed between BP and baPWV. In addition, NO increased in the group supplemented by L-citrulline, followed by increments in plasmatic L-citrulline, L-arginine and L-arginine/ADMA ratio levels. Moreover, a positive correlation between plasma L-arginine increments and baPWV reduction was also observed. These findings suggest that short-term L-citrulline supplementation may improve arterial stiffness independently of blood pressure reduction [133].

A cross-sectional clinical trial was conducted on 30 patients diagnosed with coronary artery disease and nitroglycerin-dependent flow-mediated vasodilation (FMD/NMD < 1). Subjects randomly divided into groups of 15 patients were treated with L-citrulline or a placebo for 15 days in a two-step protocol. At the end of the intervention period patient brachial artery diameters were determined by ultrasound again and compared with the data obtained before starting the treatment. The administration of L-citrulline improved the mean FMD/NMD ratio by 1.03 ± 0.09 mm (>1) and the mean FMD value by 4.96 ± 0.72 mm when compared to measures before the treatment, namely FMD/NMD ratios (0.91 ± 0.08 mm; <1) and FMD measures (4.04 ± 0.51 mm). No significant alterations were noted in the placebo group for the mean FMD/NMD ratio and mean FMD values (0.92 ± 0.09 and 4.06 ± 0.22 mm, respectively) [134].

The effects of chronic NO precursor supplementation on vascular function and exercise performance in elderly subjects from 60 to 70 years of age have also been evaluated. NO_3_^−^ and L-citrulline supplementation (N + C) were employed to activate both the NOS-independent and NOS-dependent pathways after L-arginine synthesis, as aging may decrease NO bioavailability due to NOS activity impairment and lack of NOS substrate. In this double-blind, randomized study, 24 healthy older adults, 12 males and 12 females, aged 64 ± 2 years, were evaluated through vascular function assessments and physical tests such as knee extensions and full-body exercise, as well as incremental cycling before and after the ingestion of NO precursors through salad intake containing 520 mg of NO_3_^−^ and 6 g of L-citrulline, or a placebo, taken for 30 days. The results observed following the 4-week supplementation indicated no changes in PWV measures [135]. The effectiveness of L-citrulline supplementation on endothelial dysfunction was also evaluated in 22 patients diagnosed with vasospastic angina presenting impaired brachial artery FMD (<5.5%) aged 41 to 46 years old. Capsules containing L-citrulline (800 mg/day) were administered for 8 weeks in an open-label trial. Blood samples were drawn before supplementation, 4 and 8 weeks after the beginning of supplementation, and at 4 weeks after the end of the 12-week follow-up period. Plasma NOx (nitrite + nitrate), ADMA, amino acids, hematological and biochemical markers, and serum oxidized lipids were evaluated. Endothelial function was assessed by FMD measures on the same day of blood collection. L-citrulline supplementation significantly increased plasma L-arginine concentrations at 8 weeks compared to the baseline. L-Citrulline supplementation also exerted a significant improvement in FMD at 4 and 8 weeks and maintained its effects at 4 weeks after the end of the intake. After supplementation, a marked but not significant increase in plasma NOx levels was observed [136].

When comparing the intervention periods, using L-citrulline, as an isolated compound, administered as a capsule (minimum 5.6 g of L-citrulline) it showed that a better effect can be achieved than the consumption of watermelon extract. However, when the intervention time was long—over 6 weeks—an improvement in endothelial function was observed with the supplementation of at least 6 g of L-citrulline from watermelon extract. In this way, taken together, the scientific evidence described herein support the administration of oral L-citrulline and watermelon extracts as promissory nutritional supplements to improve the cardiovascular system function. Although these studies demonstrate the potential of L-citrulline and watermelon extract to improve endothelial function, in the selected individuals, L-citrulline dose and duration and watermelon supplementation appear to have affected the magnitude of these effects, thus requiring further investigations to obtain a complete and clear scenario concerning this food and/or its bioactive compound in the cardiovascular physiology.

## 5. Potassium

K^+^ ions are the most abundant intracellular cations in living organisms, playing a role in total body fluid volume maintenance, acid–base balance, transmembrane potential establishment, electrical excitation in synapses and neuromuscular junctions and bloodstream flow, among others [137,138]. K^+^ roles in the cardiovascular system have been reported in several reviews, meta-analyses, clinical trials, and epidemiological data, pointing to an association between abnormal serum K^+^ levels and the pathophysiology of several conditions, such as hypertension, heart failure, coronary heart disease and stroke [137,139,140].

The current K^+^ intake in populations worldwide is considered to be below the optimum amount of 4700 mg/day, according to the Food and Nutrition Board of the Institute of Medicine recommendations [141]. Potassium is obtained in certain average amounts in foods such as dry fruit, nuts and seeds (7189.2 mg.kg^−1^), meat and meat products (4275.9 mg.kg^−1^), fish and seafoods (2789.1 mg.kg^−1^), cereals (2094.8 mg.kg^−1^), potatoes (4054.7 mg.kg^−1^) and tomatoes (4244.1 mg.kg^−1^). However, decreased K^+^ and increased Na^+^ intake in foods due to processing and low magnesium intakes all contribute to K^+^ intake and excretion imbalances, altering the serum levels of this ion [142,143,144].

**Figure 3:**
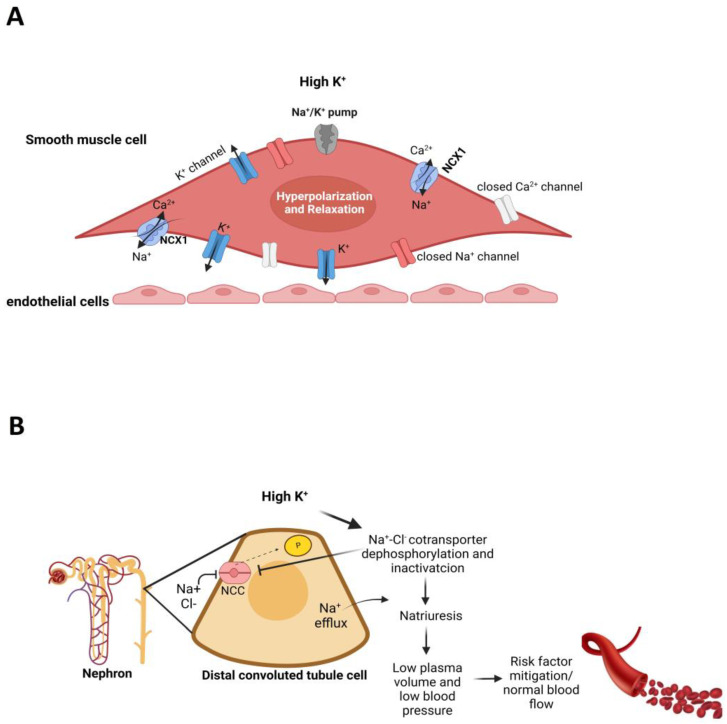
Effects of vasoactive high potassium level on vascular smooth muscle cells (**A**) and on nephrons (**B**), inducing natriuresis and reducing plasma volume. NCX1—sodium-calcium exchanger type 1, NCC—sodium-chloride cotransporter.

In the cardiovascular system, K^+^ is a vasoactive element that plays several roles with regard to vascular ECs, vessel dilation and blood flow. K^+^ stimulates the number or turnover of Na^+−^K^+^-ATPase pumps and the opening of K^+^ channels in VSMCs, resulting in hyperpolarization, inactivation of Na^+^ and Ca^2+^ channels and vasorelaxation [142,145]. In addition, Na^+^-K^+^ pump stimulation decreases intracellular Na^+^, causing the sodium-calcium exchanger type 1 (NCX1) to favor Ca^+^ efflux from cells, leading to reduced vascular tone and K^+^-mediated vasodilation [145,146] (Figure 3A). Around 98% of K^+^ ions are maintained in the intracellular compartment, but this element’s role as a physiological blood flow regulator depends on its increase in vascular beds [41,147]. Furthermore, K^+^ may support the cardiovascular system through the dephosphorylation of sodium-chloride cotransporter (NCC) in the distal convoluted tubule in nephrons, deactivating NCC and promoting Na^+^ efflux. Natriuresis reduces plasma volume and blood pressure while softening endothelial cells, increasing NO release (Figure 3B). Thus, the main reason for K^+^ supplementation is hypokalemia correction and cardiovascular abnormality prevention [140,148,149,150].

The dietary pattern of human populations is characterized by high Na^+^ intake, leading to high blood pressure and impaired endothelial function. On the other hand, increased K^+^ intake can counteract the deleterious hemodynamic effect promoted by Na^+^, which has motivated several clinical interventions to better describe dietary K^+^ effects [151,152].

A randomized crossover trial investigated the effect of meals offered once on different occasions and containing different K^+^ contents—38 mmol or 3 mmol—simultaneously to the intake of Na^+^—65 mmol or 6 mmol—on postprandial endothelial function and arterial stiffness in normotensive individuals [37]. A K^+^ intake of 38 mmol attenuated postprandial decreases in FMD following a 65 mmol Na^+^ meal. The hyperpolarizing effect on smooth cells mediated by increased K^+^ levels and NO release has been proposed as a possible underlying mechanism for the detected FMD improvement. However, no artery stiffness effects were observed.

A KCl 60 mmol intake also counteracts ambulatory endothelial dysfunction, evaluated by plasmatic endothelin-1 levels following chronic NaCl loading or a high NaCl intake of 308 mmol/7 days by 155 salt-sensitive and non-salt-sensitive individuals. Although these results were obtained by indirect measures, comprising the ambulatory arterial stiffness index (AASI) and plasma endothelin-1 levels, the findings indicate that high K^+^ intake promotes beneficial effects, reducing cardiovascular risks by protecting endothelial function [153]. After 7 days of 33 non–salt-sensitive adults following the different K^+^/Na^+^ ratio diets, ranging from low to high, FMD measures increased in individuals who followed the diet containing 65 mmol K^+^ plus 300 mmol Na^+^ (moderate) and 120 mmol K^+^ plus 300 mmol Na^+^ (high) compared to the diet containing 65 mmol K^+^ plus 50 mmol Na^+^ (low), which exhibited discrete improvements compared to baseline values, although lower than the effects observed for the higher K^+^ concentrations. It is important to note that the FMD was reduced by 23% when shifting from the low to high Na^+^ (50 to 300 mmol) diet, but was restored by the intake of 120 mmol K^+^ (3.66 ± 0.01 to 3.79 ± 0.01 mm) [154]. Other studies have also reported beneficial endothelial function effects following high K^+^ intake provided by inorganic potassium intake or high K^+^-^−^diets in both healthy and unhealthy individuals [155,156]. These results point to the promising effects of acute K^+^ supplementation on endothelial dysfunction management or prevention as conventional drug therapy adjuvants or potentiators.

In untreated pre-hypertensive and hypertensive adults, chronic 2.8 g K^+^ supplementation (71.6 mmol) for 4 weeks improved FMD by 1.16% (*p* = 0.005) in an average of 83% of the subjects compared with the placebo group in a randomized cross-over study with an entirely controlled diet. These findings have potential clinical relevance, since for every 1% increase in FMD, there is an 8–13% reduction in the risk of cardiovascular events, as previously reported [156,157,158]. On the other hand, previous studies have reported an inverse relationship between habitual K^+^ intake and PWV [159], although investigations on arterial stiffness effects followed by K^+^ supplementation independent of other variables have demonstrated poor or null benefits [160]. The acute administration of moderate or high K^+^ diets resulted in no PWV differences following 6 days of supplementation in 35 healthy subjects with 80 (3.11 g) or 150 mmol (5.69 g) of K^+^ [150]. In the chronic regimen, the supplementation of 21 healthy individuals with 100 mmol K^+^ for 28 days led to a discrete decrease in arterial stiffness from 5.9 m/s to 5.6 m/s compared to the placebo treatment (*p* = 0.031) [161]. In another 6-week clinical trial, a dietary intervention where the effects of placebo capsules containing 20 or 40 mmol of K^+^ from fruits and vegetables was compared to the ingestion of 40 mmol potassium citrate, with no effects on PWV measures in 48 subjects displaying early hypertension [160]. Even higher K^+^ doses of 60 mmol (4.8 g) administered for 6 weeks to 40 patients with increased cardiovascular risk did not show change on baseline K^+^ and had no influence on PWV [162]. These studies together indicate no considerable effects of K^+^ on arterial stiffness following supplementation through K^+^-diets containing between 20 mmol and 150 mmol.

To the best of our knowledge, a randomized, double-blind, placebo-controlled crossover 4-week trial in 42 untreated mildly hypertensive subjects is one of the few exceptions, where the ingestion of K^+^ salts, potassium chloride or potassium bicarbonate at 64 mmol equally improved the carotid–femoral PWV [163], although the evidence for the role of K^+^ on arterial stiffness is weak. This was confirmed by the meta-analysis data from randomized controlled trials reported by Tang and Liu [164], which revealed no significant arterial stiffness improvement following K^+^ supplementation. The authors described the pooled evidence as conflicting, due to several factors, such as age and gender variabilities, where older people and men were more likely to achieve decreased PWV. The small sample size of the studies, with fewer than 50 individuals in most of them, the short-time interventions of less than 6 weeks, and the limited number of available clinical reports does not allow for a clear indication that K^+^ therapy can improve arterial stiffness in individuals at risk for cardiovascular events; however, K^+^ supplementation even under these limitations acutely exhibited a protective role on endothelial function according to the presented literature (Table 4).

**Figure 4:**
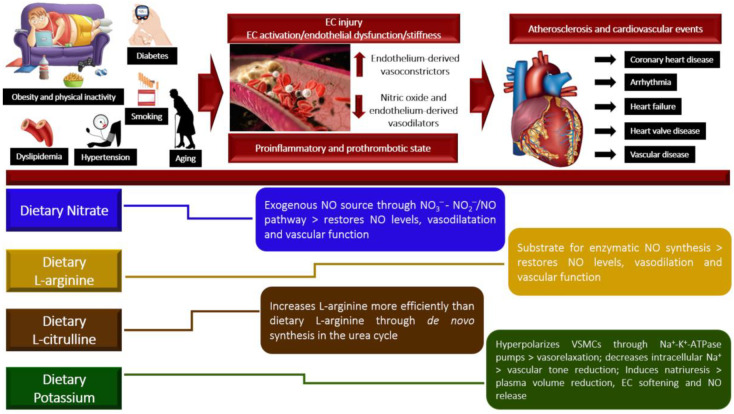
Brief representative scheme of the mechanisms by which risk factors damage vascular function predisposing to cardiovascular events and the underlying pathways of dietary vasoactive compounds on vascular physiology.

Taken together, the main mechanisms by which the dietary NO_3_^−^, L-arginine, L-citrulline and K^+^, in their pure forms or as a part of rich-food matrices, exert their effects on arterial hemodynamics, are presented in Figure 4, which includes the pathophysiological mechanisms that culminate in the low availability of nitric oxide and the pathway of action of the four vasoactive compounds on cardiovascular function, showing how these dietary interventions can benefit or overcome the endothelium dysfunction and modulate arterial stiffness, in order to benefit individuals mainly those with hypertension and other risk factors for cardiovascular diseases.

## 6. Conclusions

Cardiovascular diseases are the leading cause of death worldwide, and many of them occur prematurely. Therefore, early diagnosis and treatment can contribute to reducing morbidity and mortality rates resulting from untreated cardiovascular diseases. This narrative review reinforces the relevance of dietary interventions in improving endothelial dysfunctions and arterial stiffness, reflected by the FMD and PWV, noninvasive clinical techniques recognized by the scientific community as vascular event predictors. Different classes of compounds found in food matrices, such as the amino acids L-arginine and L-citrulline, the mineral K^+^ and the anion NO_3_^−^, can positively interfere with endothelium and artery physiology, even when associated with other unhealthy diets and lifestyles that negatively affect vascular homeostasis, such as high sodium intake or physiopathological conditions like kidney disease, heart diseases, high fat intake, obesity, smoking and aging. This critical review indicates that L-arginine, L-citrulline, K^+^ and NO_3_^−^ exhibit pronounced effects on FMD following mainly chronic interventions, particularly NO_3_^−^ found in high-nitrate beetroot formulations, whose effects have been clearly demonstrated and seem to be irrefutable. A minimum dosage of 370 mg (6.0 mmol) of NO_3_^−^ present in ~250 mL of juice, ~40 g of beetroot cereal bar, or ~100 g of beetroot gel, for example, for at least 4 weeks should be considered as an initial regimen for non-medicinal therapy. Isolated L-citrulline is effective for restoring vascular function even acutely when administered at doses of 5.6 g. However, to obtain the same effect through the ingestion of watermelon does not seem to be advantageous due to the low concentration of L-citrulline in the fruit, requiring large serving portions combined with the need for a long intervention time. L-arginine is probably the most studied vasoactive dietary supplement, because it is a direct biosynthetic precursor of NO, and doses between 4.5 and 21 g, which can probably only be achieved by the intake of capsules containing the dietary supplement, have shown consistent effects on endothelial function. On the other hand, the effects and tests carried out to evaluate the effectiveness of K^+^ on artery stiffness are still limited, and satisfactory physiological effects have been shown only on endothelial function, which can be obtained following the intake of this vasoactive compound at a dose of 1.5 g/day (38 mmol/day), which can be achieved by adopting the Mediterranean diet, which is characterized by regular consumption of a variety of vegetables, fruits, grains and white meats. Clinical trials including longer supplementation periods, subjects displaying uniform metabolic conditions and larger sample sizes are paramount to improve data on K^+^ supplementation due to the importance of this dietary intervention in the prevention of cardiovascular events. Finally, this review unequivocally demonstrates that L-arginine, L-citrulline, K^+^ and NO_3_^−^ can be considered low-cost non-drug therapies, comprising simple and no-risk alternatives to improve cardiovascular function. These vasoactive compounds naturally found in food matrices display the potential to be administered in their pure form or as enriched formulations to increase bioactive compound concentrations in foodstuffs associated with drug therapies acting synergically in the same or in different pathways, but safely and contributing to decreased cardiovascular pathologies.

## Figures and Tables

**Table 1:** Selected clinical trials following NO_3_^−^ supplementation: dose, intervention period, clinical trial design and main outcomes.

NO_3_^−^ Content/Vehicle	Subjects/Age	Intervention Period	Experimental Design	Main Outcomes	Study
≈8.0 mmol/140 mL of beetroot juice	20 healthy overweight and slightly obese individuals (61.0 ± 7.0 y)	Single dose	Double-blind Randomized Placebo-controlled Crossover	↑ NOx ↓ DBP ↑ FMD PWV, AIx and central AIx—no effects	Joris et al. [78]
3.55 mmol/250 g of cooked spinach	26 healthy subjects (58.8 ± 7.6 y)	Single dose	Double-blind Randomized Placebo-controlled Crossover	↑ salivary NOx ↑ large artery elasticity index ↓ PP, SBP, cardiac ejection time, cardiac output, stroke volume and total vascular impedance	Liu et al. [79]
9.4 mmol/500 mL of beetroot juice	26 healthy adults (young: 25 ± 4 y, n = 14; older: 64 ± 5 y, n = 12)	Single dose	Double-blind Randomized Placebo-controlled Crossover	↑ plasma NO_2_^−^ ↓ Peripheral and aortic SBP, DBP, MAP and aPP in young and older subjects to a similar degree. ↓ AIx in young adults AIx older adults no—effect	Hughes et al. [80]
9.7 mmol/140 mL of beetroot juice	13 healthy postmenopausal women (22.0 ± 1.0 y)	Single dose	Double-blind Randomized Placebo-controlled Crossover	↑ plasma NOx ↓ brachial and aortic SBP, DBP and MAP Vascular functions—no effects	Kim et al. [81]
13 mmol/140 mL of beetroot juice	15 healthy older men (69 ± 4 y)	Single dose	Double-blind Randomized Crossover Placebo-controlled Acute ingestion	↑ plasma NOx BP and PWV—no effect ↑ FMD ↓ AIx75	Walker et al. [82]
12.9 mmol/140 mL of beetroot juice	165 subjects COPD grade II–IV. 78 in nitrate-rich group (70 ± 14 y) and 87 in placebo group (68 ± 13 y).	Single dose	Double-blind Randomized Placebo-controlled Parallel group	↑ Exercise capacity ↓ SBP, DBP and MAP ↑ FMD	Pavitt et al. [83]
6.20 mmol/70 mL of beetroot juice	Study 1: 13 hypertensive subjects taking antihypertensive medications (age: 53 ± 12 y) Study 2: 14 hypertensive subjects not taking antihypertensive medications (49 ± 13 y)	3 days	Double-blinded Randomized Placebo-controlled Acute-ingestion	Study 1: ↑ plasma NOx ↓ plasma L-arginine Blood pressure and vascular function—no effects Study 2: ↑ plasma NOx ↑ plasma L-arginine ↓ SBP, DBP and MAP ↑ FMD	Broxterman et al. [84]
6.2 mmol/70 mL of beetroot juice	11 healthy male subjects (age 30 ± 7 y)	1 week	Single-blinded Randomized Crossover Placebo-controlled	↑ plasma and salivary NOx Blood pressure and vascular function—no effect	Burleigh et al. [51]
6.4 mmol/250 g of green leafy vegetables (spinach, lettuce, spinach, rocket, and other leafy greens)	38 healthy subjects	1 week	Randomized Placebo-controlled Crossover	↑ plasma NOx SBP and DBP—no effects PWV and AIx—no effects	Bondono et al. [70]
NO_3_^−^: ≈7.0 mmol/140 mL of beetroot juice	27 hypertensive and older subjects (63.2 ± 4.4 y)	1 week	Double-blind Randomized Placebo-controlled Crossover	↑ urinary, salivary and plasmatic NOx Home BP, SBP and DBP 24 h ambulatory—no effects	Bondono et al., [71]
7.5 mmol/250 mL of beetroot juice	27 subjects with type 2 diabetes (67.2 ± 4.9 y)	2 weeks	Double-blind Randomized Placebo-controlled Crossover	↑ NOx FMD—no effect Insulin sensitivity—no effect SBP, DBP and MAP—no effects Microvascular endothelial function—Perfusion response—Laser Doppler—no effect	Gilchrist et al. [69]
250 mL of beetroot juice (NO_3_^−^ contents not specified) 250 g cooked beetroot (NO_3_^−^ contents not measured)	24 hypertensive subjects without medication (55.2 ± 11.4 y beetroot juice group, 53.3 ± 10.3 y cooked beetroot group)	2 weeks	Assessor-blind Randomized Crossover	↓ ICAM-1, VCAM-1 and E-selectin by both treatments ↓ hs-CRP, TNF-α and IL-6 by both treatments ↓ TAC after beetroot juice ↓ SBP and DBP by both treatments ↑ FMD (beetroot juice > cooked beetroot)	Asgary et al. [85]
6.45 mmol/70 mL of beetroot juice	20 older subjects (65 ± 8 y)	2 weeks	Double-blinded Randomized Placebo-controlled	↑ plasma NO_3_^−^ ↓ SBP and DBP ↑ FMD	Jones et al. [86]
9.57 mmol/60 g of beetroot-cereal bar	5 patients displaying three risk factors for CVD (54.25 ± 4.64 y)	3 weeks	Double-blind Randomized Placebo-controlled Crossover	↑ plasma NOx ↓ SBP and DBP ↓ PWV, AP, AIx, _ao_SP, _ao_PP and arterial age ↓ endothelial dysfunction by improvements in CVC peaks and AUC	Baião et al. [87]
≈6.4 mmol/250 mL of beetroot juice	34 drug-naive and 34 treated hypertensive subjects (57.6 ± 13.9 y)	4 weeks	Double-blind Randomized Placebo-controlled	↑ plasma NOx ↑ plasma cGMP ↓ 24 h BP ↓ PWV and AIx ↑ FMD and time to peak	Kapil et al. [72]
NaNO_3_^−^: 10.5 mmol/supplement dissolved in drinking water	11 healthy older subjects (63.0 ± 5.0 y)	4 weeks	Double-blind Randomized Placebo-controlled	↑ NO synthesis (through plasma NO_3_^−^ and NO_2_^−^) ↓ SBP, DBP, PWV and AIx ↑ FMD	Rammos et al. [73]
6.0 mmol/250 mL of beetroot juice	65 hypercholesterolemic subjects (53.3 ± 10.1 y)	6 weeks	Double-blind Randomized Placebo-controlled Parallel group	↑ plasma, salivary and urinary NOx ↓ platelet-monocyte aggregates ↓ SBP DBP and HR—no effects ↓ Aix ↓ aPWV ↑ FMD	Velmurugan et al. [88]
4.03 mmol/beetroot juice	37 subjects with type 2 diabetes mellitus	8 weeks	Double-blinded Randomized Placebo-controlled parallel group Chronic ingestion	↑ plasma NO_2_^−^ ↓ Peripheral SBP ↓ Central SBP and AP ↓ MAP ↓ AIx	Bock et al. [89]

↑increase; ↓decrease; AIx, augmentation index; AIx75, augmentation index adjusted to heart rate 75 bpm; _ao_PP, aortic pulse pressure; _ao_SP, aortic systolic pressure; AP, augmentation pressure; AUC, area under the perfusion curve; CAD, coronary artery disease; cGMP, cyclic guanosine monophosphate; DBP, diastolic blood pressure; COPD, chronic obstructive lung disease; CVC, cutaneous microvascular conductance; FMD, mediated flow dilatation; HR, heart rate; hs-CRP, high-sensitivity C-reactive protein; ICAM-1, intercellular adhesion molecule 1; IL-6, interleukin 6; MAP, mean arterial pressure; NOx, nitrate and nitrite concentrations; NO_2_^−^, nitrite; NO_3_^−^, nitrate; PP, pulse pressure; PWV, pulse wave velocity; aPWV, aortic pulse wave velocity; SBP, systolic blood pressure; TAC, total antioxidant capacity; TNF-α, tumour necrosis factor alpha; VCAM-1, vascular cell adhesion molecule 1; y, years.

**Table 2:** Selected clinical trials considering administered L-arginine capsules, supplemented dose, intervention period, experimental design, and main outcomes.

L-Arginine Content/Vehicle	Subjects/Age	Intervention Period	Experimental Design	Main Outcomes	Study
16 g/in capsules	12 healthy older (8 males, 4 females) (73.8 ± 2.7 y)	2 weeks	Double-blind Randomized Placebo-controlled Crossover	↑ plasma L-arginine ↑ FMD	Bode-Boger et al. [34]
21 g/in capsules	10 CAD males (41 ± 2 y)	10 days	Double-blind Randomized Placebo-controlled Crossover	↑ plasma l-arginine ↑ FMD BP and HR—no effects	Adams and Celermajer [99]
6 g/in capsules	10 smokers (3 males, 7 females) (24.4 ± 0.95 y)	3 occasions after acute smoking	Double-blind Randomized Placebo-controlled Crossover	↑ FMD ↓ cfPWV and Alx	Siasos et al. [97]
21 g/in capsules	12 smokers (5 males, 7 females) (24.4 ± 0.95 y)	three occasions after acute smoking	Double-blind Randomized Placebo-controlled Crossover	FMD—no effects ↓ cfPWV and Alx	Siasos et al. [98]
10 g/in capsules	33 CAD subjects (21 males, 12 females) (58 ± 7 y)	4 weeks	Open-label Randomized Crossover	↑ FMD ↓LDL oxidation ICAM-1, VCAM-1 and P-seletin—no effects	Yin et al. [101]
6.4 g/in capsules	64 CAD subjects (65 ± 10 y)	6 months	Double-blind Placebo-controlled	↑ plasma l-arginine ↑ c-GMP ↓ ADMA ↑ reactive hyperemia	Lucotti and Piatti [102]
8 g/in capsules	28 PCOS women (24.3 ± 3.5 y)	4 weeks	Double-blind Randomized Placebo-controlled	↑ plasma NOx ↑ reactive hyperemia Blunted ↑ BP-drospirenone induced	Battaglia et al. [104]
3 g/in capsules	30 CHF subjects (17 male, 13 female) (63 ± 14.5 y)	8 weeks	Double-blind Randomized	↓ MAT/TT ratio after forearm occlusion	Orea-Tejeda et al. [105]
9 g/in capsules	30 CKD and high PWV subjects (24 males, 6 females) (49.4 ± 11)	12 weeks	Randomized Open-label	↑ plasma Nox ↓ cfPWV, crPWV, Alx and AP	Annavarajula et al. [107]
4.5 g/in capsules	36 overweight (22 males, 13 females) (45 ± 8.9 y)	4 weeks	Double-blind Randomized Placebo-controlled Crossover	↑ plasma L-arginine Blunted ↓FMD high fat-induced ↑ reactive hyperemia	Deveaux et al. [108]

↑ increase; ↓ decrease; AASI, ambulatory arterial stiffness index; ADMA, asymmetric dimethylarginine; AIx, augmentation index; AP, aortic augmentation pressure; BP, blood pressure; CAD, coronary artery disease; cfPWV, carotid–femoral pulse wave; crPWV, carotid-radial pulse wave; cGMP, cyclic guanosine monophosphate; CHF, congestive heart failure; CKD, chronic kidney disease; FMD, Flow-mediated dilation; HR, heart rate; ICAM-1, intercellular adhesion molecule-1; MAT, maximum amplitude time; NOx nitrate plus nitrite concentrations; PCOS, Polycystic ovary syndrome; PWV, pulse wave velocity; TT, total time of the curve; VCAM-1, vascular cellular adhesion molecule-1; y, years.

**Table 3:** Selected clinical trials on L-citrulline/watermelon supplementation, dose, intervention period, experimental design and main outcomes.

L-Citrulline Content/Vehicle	Subjects/Age	Intervention Period	Experimental Design	Main Outcomes	Study
watermelon rind (5 g) watermelon flesh (5 g) watermelon rind (0.1 g)	6 overweight/obese subjects (32.2 ± 7.6 y)	1 week	Randomized Placebo-controlled Crossover	FMD—no effect	Fan et al. [125]
4 g/30 g of micro-encapsulated watermelon	11 healthy adults	3 occasions with a 1 week interval	Randomized Single-blind Crossover Placebo-controlled	↑ FMD ↑ plasma L-citrulline and L-arginine	Volino-Souza et al. [126]
500 mL watermelon juice	17 healthy young adults (21–25 y) (6 males/11 females)	2 weeks	Randomized Placebo-controlled Double-blind Crossover	↑ FMD ↑ plasma L-arginine	Vincellette et al. [127]
1.63 g/Two servings of 360 mL of 100% watermelon juice	21 healthy postmenopausal women (55–70 y)	4 weeks	Randomized Double-blind Placebo-controlled Crossover	FMD, PWV, MAP—no effects SBP and DBP—no effects	Ellis et al. [128]
watermelon (6 g/d L-citrulline/L-arginine)	12 postmenopausal women (57 ± 1 y)	6 weeks	Randomized Placebo-controlled Crossover	↓ baPWV ↓ SBP and DBP ↓ aortic SBP2 radial SBP2—no effect aortic and radial—no effects	Figueroa et al. [129]
2.7 g/in watermelon	9 pre-hypertensives subjects (4 male/5 female) (54 ± 3 y)	6 weeks	Randomized Placebo-controlled	↓ AIx cfPWV—no effect	Figueroa et al. [130]
watermelon (containing L-citrulline 1.3 g plus L-arginine 2.7 g)	14 adults (11 female/3 male) (58 ± 1 y) prehypertensive or stage 1 hypertension	6 weeks	Randomized Placebo-controlled Two-period Crossover	↓ cAIx ↓ SBP, DBP, MAP HR and ABI—no effects	Figueroa et al. [119]
10 g/L-citrulline capsules	7 older HF adults (>60 y) and 7 healthy young subjects (21–40 y)	2 days	Kinetic study Placebo-controlled	↑ plasma L-arginine (in older adults) ↑ NO synthesis rate (in older adults) ↑ NO synthesis rate (in older adults) RH-FBF—unaffected	Kim et al. [131]
6 g/citrulline capsules	25 sedentary hypertensive postmenopausal women (50–4 y)	4 weeks	Double-blind, Randomized Placebo-controlled	↑ plasma L-arginine ↑ FMD ↑ aortic DBP and MAP cfPWV—no effects brachial BP—no effects	Maharaj et al. [132]
5.6 g/L-citrulline capsules	15 healthy subjects (58.3 ± 4.4 y)	1 week	Double-blind Randomized Placebo-controlled Parallel-group	↓ baPWV DBP and SBP—no effects ↑ NO ↑ NOx ↑ plasma L-citrulline ↑ plasma L-arginine ↑ plasma ratio of arginine/ADMA ↑ endogenous inhibitor of NO synthase	Ochiai et al. [133]
100 mg/kg body weight in capsules	30 CAD and FMD/NMD (<1) subjects	2 weeks	Randomized Crossover placebo-controlled	↑ FMD/NMD ↑ FMD	Safi et al. [134]
6 g/citrulline drink	24 healthy subjects (12 males/12 females) (64 ± 2 y)	4 weeks	Double-blind Randomized	↓ SBP and DBP PWV—no effect NO—no effect	Roux-Mallouf et al. [135]
800 mg/L-citrulline capsules	22 diagnosed vasospastic angina patients (41–46 y)	8 weeks	Open label	↑ plasma NOx ↓ ADMA ↑ FMD ↑ plasma L-arginine/ADMA ratio	Morita et al. [136]

↑increase; ↓decrease; aAIx, aortic augmentation index; ABI, ankle–brachial index; AIx, augmentation index; baPWV, brachial–ankle pulse wave velocity; CAD, coronary artery disease; cAIx, carotid augmentation index; DBP, diastolic blood pressure; FMD, mediated flow dilatation; HF, heart failure; HR, heart rate; MAP, mean arterial pressure; NO, nitric oxide; NOx, nitrate plus nitrite concentrations; PWV, pulse wave velocity; RH-FBF, reactive hyperemic forearm blood flow; SBP, systolic blood pressure; y, years.

**Table 4:** Selected clinical trials following potassium supplementation associated or not to Na^+^ supplementation, dose, intervention period, experimental design and main outcomes.

K^+^ Content/Vehicle	Subjects/Age	Intervention Period	Experimental Design	Main Outcomes	Study
Diet containing: (1) K^+^—3 mmol + Na^+^—3 mmol (2) K^+^—3 mmol + Na^+^—65 mmol (3) K^+^—38 mmol + Na^+^—65 mmol	34 healthy subjects (16 males, 18 females) (37 ± 15 y)	1 day	Double-blind Randomized Crossover	↑ FMD (3) ↓ AIx (1) (2) (3) cfPWV—no effect BP—no effect	Blanch et al. [37]
Diet containing: (1) K^+^ 80 mmol (2) K^+^ 150 mmol	35 healthy subjects (26 males, 9 females) (31 ± 11 y)	6 days	Single-blind Randomized Crossover	↑ FMD at 150 mmol cfPWV and AIx—no effect SBP and DBP—no effect ADMA, ICAM-1 and Endothelin-1—no effect	Blanch et al. [155]
K^+^—60 mmol + Na^+^—308 mmol in meals	155 healthy subjects (89 males, 66 females) (52.7 ± 10 y)	1 week	Randomized Open-label	↓ AASI ↓ Endothelin-1 ↓ SBP and DBP	Liu et al. [153]
Diet containing: (1) K^+^—65 mmol + Na^+^—50 mmol (2) K^+^—65 mmol + Na^+^—300 mmol (3) K^+^ 120 mmol + Na^+^ 300 mmol	33 healthy subjects (16 males, 17 females) (27 ± 1 y)	1 week	Randomized Open-label	↑ K^+^ excretion (1) ↑ FMD (1)(3) > (2) cfPWV and AIx—no effect SBP and DBP—no effect	Smiljanec et al. [154]
71.6 mmol/2.8 g K^+^ capsules	36 untreated (pre) hypertensive subjects (24 males, 12 females (65.8 ± 8.8 y)	4 weeks	Double-blind Randomized Placebo-controlled Crossover	↑ FMD ↓ IL-8	Gijsbers et al. [156]
100 mmol/K^+^ capsules	21 healthy subjects (9 males, 12 females) (26 ± 15.5 y)	4 weeks	Randomized Placebo-controlled Crossover	↑ cfPWV AIx no effect 24 h-BP—no effect CBP—no effect	Matthesen et al. [161]
Diet containing: (1) placebo + 20 mmol K^+^ from foods (2) K^+^—20 mmol in meals (3) K^+^—40 mmol in meals (4) K^+^—40 mmol/capsules alone	48 early hypertensive subjects (16 males, 13 females) (45 ± 0.49 y)	6 weeks	Single-blind Randomized Placebo-controlled Crossover	K^+^ excretion—no effect FMD, cfPWV—no effect SBP and DBP—no effect	Berry et al. [160]
60 mmol/K^+^ capsules	40 high CVD risk subjects (32 males, 8 females) (54.8 ± 1.1 y)	6 weeks	Double-blind Randomized Placebo-controlled Crossover	crPWV and AIx—no effects ↓ SBP and DBP ↑ plasma renin and aldosterone	Graham et al. [162]
(1) KCl—64 mmol/capsules (2) KHCO_3_—64 mmol/capsules	Untreated mildly hypertensive subjects (30 males, 12 females) (51 ± 10 y)	12 weeks	Double-blind Randomized Placebo-controlled Crossover	↑ K^+^ excretion (1) (2) ↑ FMD (1) (2) ↑ cfPWV (1) (2) ↓ SBP (1)	He et al. [163]

↑increase; ↓decrease; AIx, augmentation index; aPWV, aortic pulse wave velocity; cfPWV, carotid-femural pulse wave velocity, crPWV, carotid-radial pulse wave velocity; CBP, central blood pressure; DBP, diastolic blood pressure; FMD, mediated flow dilatation; ICAM-1, intercellular adhesion molecule 1; IL-6, interleukin 6; HR, heart rate; KCl, potassium chloride; KHCO_3_, potassium bicarbonate; SBP, systolic blood pressure; VCAM-1, vascular cell adhesion molecule 1; y, years.

## Data Availability

Data that support the findings of these experiments are available upon request.

## Abbreviations

AASI, ambulatory arterial stiffness index; aAIx, aortic augmentation index; ABI, ankle–brachial index; ADMA, asymmetric dimethylarginine; AIx, augmentation index; AIx75, augmentation index adjusted to heart rate 75 bpm; ALDH, aldehyde dehydrogenase; AO, aldehyde oxidase; aoPP, aortic pulse pressure; aoSP, aortic systolic pressure; AP, augmentation pressure; AUC, area under the perfusion curve; baPWV, brachial–ankle pulse wave velocity; BH_4_, tetrahydrobiopterin; BMI, body mass index; BP, blood pressure; CA, carbonic anhydrase; CAD, coronary artery disease; Ca^2+^, calcium; cAIx, carotid augmentation index; cfPWV, carotid–femoral pulse wave velocity; crPWV, carotid-radial pulse wave; cGMP, cyclic guanosine monophosphate; CHF, congestive heart failure; CKD, chronic kidney disease; COPD, chronic obstructive lung disease; CVC, cutaneous microvascular conductance; CVD, cardiovascular disease; DBP, diastolic blood pressure; deoxyHb, deoxyhemoglobin; deoxyMb, deoxymyoglobin; ECs, endothelial cells; eNOS, endothelial nitric oxide synthase; FAD, flavin adenine dinucleotide; FAO, Food and Agriculture Organization; FMD, flow-mediated vasodilation; FMD/NMD, nitroglycerin-dependent flow-mediated vasodilation; FMN, flavin monocleotide; GC, guanylate cyclase; GTP, guanosine triphosphate; HDL, high density lipoprotein; HNO_2_, nitrous acid; HR, heart rate; hs-CRP, high-sensitivity C-reactive protein; ICAM-1, intercellular adhesion molecule 1; IL-6, interleukin 6; iNOS, inducible nitric oxide synthase; K^+^, potassium; KCl, potassium chloride; KHCO_3_, potassium bicarbonate; LDL, low density lipoprotein; MAT, maximum amplitude time; MAP, mean arterial pressure; MetHba, metglobinemia; mm Hg, millimeter of mercury; Na^+^, sodium; NaCl sodium chloride; NAD, nicotinamide adenine dinucleotide; NADPH, nicotinamide adenine dinucleotide phosphate; NCC, sodium-chloride cotransporter; NCX1, sodium-calcium exchanger type 1; nNOS, neuronal nitric oxide synthase; NO, nitric oxide; NO^+^, nitrosonium; NOS, nitric oxide synthase; NOx, nitrate and nitrite plasmatic levels; NO_2_, nitrogen dioxide; NO_2_^−^, nitrite; NO_3_^−^, nitrate; N_2_O_3_, dinitrogen trioxide; O_2_, oxygen; ox-LDL, oxidized-low density lipoprotein; oxyHb, oxyhemoglobin; PCOS, polycystic ovary syndrome; PP, pulse pressure; PPi, pyrophosphate; PWV, pulse wave velocity; RH-FBF, reactive hyperemic forearm blood flow; ROS, reactive oxygen species; SBP, systolic blood pressure; TAC, total antioxidant capacity; TNF-α, tumour necrosis factor alpha; TT, total time; VCAM-1, vascular cell adhesion molecule 1; VSMCs, vascular smooth muscle cells; WHO, World Health Organization; XOR, xanthine oxidoreductase; y, years.
